# Type 2 diabetes mellitus and cognitive decline in older adults in Germany – results from a population-based cohort

**DOI:** 10.1186/s12877-022-03151-y

**Published:** 2022-05-26

**Authors:** Kun Xie, Laura Perna, Ben Schöttker, Matthias Kliegel, Hermann Brenner, Ute Mons

**Affiliations:** 1 Division of Clinical Epidemiology and Aging Research, German Cancer Research Center (DKFZ), Heidelberg, Germany; 2 Medical Faculty Heidelberg, Heidelberg University, Heidelberg, Germany; 3 Department of Translational Research in Psychiatry, Max Planck Institute of Psychiatry, Munich, Germany; 4 Network Aging Research (NAR), Heidelberg University, Heidelberg, Germany; 5 Department of Psychology, University of Geneva, Geneva, Switzerland; 6 Faculty of Medicine and University Hospital Cologne, University of Cologne, Kerpener Str. 62, 50937 Cologne, Germany

**Keywords:** Type 2 diabetes mellitus, Cognitive function, Cognitive decline, Vascular risk factors

## Abstract

**Background:**

A large body of evidence supports a link between type 2 diabetes mellitus (T2DM) and cognitive function, including dementia. However, longitudinal studies on the association between T2DM and decline of cognitive function are scarce and reported mixed results, and we hence set out to investigate the cross-sectional and longitudinal association between T2DM and global as well as domain-specific cognitive performance.

**Methods:**

We used multivariable regression models to assess associations of T2DM with cognitive performance and cognitive decline in a subsample of a population-based prospective cohort study (ESTHER). This subsample (*n* = 732) was aged 70 years and older and had participated in telephone-based cognitive function assessment (COGTEL) measuring global and domain-specific cognitive performance during the 5- and 8-year follow-up.

**Results:**

Total COGTEL scores of patients with prevalent T2DM were 27.4 ± 8.3 and 29.4 ± 8.7 at the 5- and 8-year measurements, respectively, and were roughly two points lower than those of T2DM-free participants after adjustment for age and sex. In cross-sectional models, after adjustment for several potential confounders, performance in verbal short-term and long-term memory tasks was statistically significantly lower in participants with T2DM, but the association was attenuated after further adjustment for vascular risk factors. The difference in total COGTEL scores reflecting global cognitive function by T2DM status after full adjustment for confounders and vascular risk factors was equivalent to a decrement in global cognitive function associated with a four-year age difference. In longitudinal models, a statistically significantly stronger cognitive decline in patients with T2DM was observed for working memory.

**Conclusions:**

In this sample of older individuals, T2DM was associated with worse performance and stronger decline in a cognitive function test. Memory-related domains were found to be particularly sensitive to T2DM. Further large-scale prospective studies are needed to clarify potential T2DM-related predictors of cognitive decline and possible consequences on the abilities to perform patient self-management tasks in diabetes care.

**Supplementary Information:**

The online version contains supplementary material available at 10.1186/s12877-022-03151-y.

## Introduction

In 2019, 11% of women and 12.3% of men in Germany had a documented diagnosis of diabetes [1], which is among the highest prevalence figures in Europe [2]. Type 2 diabetes mellitus (T2DM) accounts for about 90% of the total diabetes cases and its prevalence increases with age [3]*.* According to an analysis of statutory health insurance data, both the prevalence and absolute case numbers of T2DM in Germany are expected to rise substantially over the next decades [4].

Cognitive impairment, which is a further challenge in aging societies, is increasingly recognized as an important complication of T2DM [5]. A recent meta-analysis estimated a prevalence of mild cognitive impairment in T2DM patients of 45% [6]. Effects of T2DM on cognitive function are of particular interest given the important role of patient self-management in diabetes care [5].

A previous study from this group found that diabetes predicted worse performance in a cognitive test five years later [7]. However, while a number of cross-sectional and cohort studies have demonstrated a link between T2DM and cognitive impairment and risk of dementia [8], comparatively few studies have examined the longitudinal association with cognitive outcomes, particularly with regards to changes in functioning of different cognitive domains, and results are heterogeneous [9]. While some longitudinal studies showed a stronger decline of cognitive functioning in older people with T2DM [10–13], others have not found statistically significant differences in cognitive decline between older adults with T2DM and those free of T2DM [14–16].

For this work, we used a subsample of a population-based cohort of older adults in Germany who had repeatedly participated in a cognitive assessment using a comprehensive neuropsychological test instrument, in order to investigate if and to what extent T2DM is associated with global as well as domain-specific cognitive performance and cognitive decline.

## Methods

### Study population

Participants in this study were a subpopulation of the on-going ESTHER prospective cohort study [17, 18]. Briefly, 9,940 participants aged 50–74 years were recruited in 2000–2002 at baseline, during a routine health check-up by their general practitioners (GPs) in the federal state of Saarland, Germany. Self-administered questionnaires were used to collect socio-demographic, lifestyle, and health-related information. The GPs also completed a questionnaire related to ESTHER participants, collected blood samples and provided medical records. The ESTHER study was approved by the ethics committee of the Medical Faculty of Heidelberg University (no. 58/2000, from May 15, 2000) and by the Medical Board of Saarland, and is being conducted in adherence with the Declaration of Helsinki. All participants provided written informed consent.

At the 5-year follow-up of the ESTHER study, conducted in 2005–2007, participants aged 70 years and older were invited to participate in a telephone-based assessment of cognitive function (COGTEL), as described in detail elsewhere [19]. In total, 1,945 eligible participants were included. Among those who participated in the cognitive assessment in the 5-year follow-up measurement (which served as our first cognitive assessment in this study), 777 participants took part in another repeated cognitive assessment using the same instrument at the 8-year follow-up in 2008–2010. For this study, the subsample of participants who had taken part in the COGTEL interviews both at 5- and 8-year follow-up were eligible for analysis. After additional exclusion of 45 participants with invalid COGTEL at first or second assessment, 732 remained for analysis. A comparison of characteristics of participants with repeated COGTEL assessment and those who were lost to the second assessment is shown in the Supplemental Table and indicates that the sample with repeated assessment had a higher share of participants with higher education and somewhat lower prevalence of T2DM, obesity, stroke, hypertension, CHD, depression and sleeping disorder.

### Cognitive function assessment

The development and validation of the COGTEL instrument has been described in detail elsewhere [20, 21]. The COGTEL version A, including tests of prospective memory, verbal short-term memory, verbal long-term memory, working memory, verbal fluency and inductive reasoning sub-components, was applied in both assessments. Individual domain-specific tasks generally originate from neuropsychological instruments with confirmed validity and reliability. In terms of the scoring system of the instrument, the scores of the sub-components were used individually or as total COGTEL score reflecting global cognitive function. The total COGTEL score was calculated as weighted sum (7.2*prospective memory + 1.0*verbal short-term memory + 0.9*verbal long-term memory + 0.8*working memory + 0.2*verbal fluency + 1.7*inductive reasoning) [21].

### Exposure and covariates

Data on diagnosis of T2DM was collected at both COGTEL measurements through a standardized questionnaire for participants, but at both time-points, more than 90% of prevalent cases were physician-validated. Participants were divided into two subgroups according to T2DM status (T2DM and T2DM-free).

Sociodemographic variables relevant to this study (age, sex and school education) were taken from the ESTHER baseline examination in 2000–2002. Lifestyle-related variables (weight, smoking status, alcohol consumption, sleeping disorder) were self-reported by participants in standardized questionnaires at the time of the first COGTEL measurement at 5-year follow-up. Body mass index (BMI) was calculated as weight divided by the square of the body height from self-reported weight at 5-year follow-up and height measured by GPs at ESTHER baseline. Disease history (stroke, hypertension, coronary heart disease, depression) was also self-reported but validated (with exception of depression) using information collected via standardized questionnaires from GPs of participants. Prevalence of hearing impairment was self-reported by participants at the COGTEL interview. HbA_1c_ was measured in full blood samples collected during the health screening exam at ESTHER baseline using high performance liquid chromatography (Bio-Rad Variant II, Bio-Rad, Munich/Germany). The *APOE* genotype was measured using TaqMan SNP genotyping assays with genotypes analyzed in an endpoint allelic discrimination read using a PRISM 7000 Sequence detection system (Applied Biosystems), and was categorized according to *APOE* e4 allele carrier status.

### Statistical analysis

Firstly, bivariate analyses were conducted to compare participants’ characteristics between those with and without T2DM at first cognitive measurement. Secondly, multiple linear and logistic regression models were conducted to estimate the age- and sex-adjusted difference in sub-component and total COGTEL scores between participants with and without T2DM at first and second cognitive measurement. Thirdly, multiple linear regression and logistic regression were performed to investigate the association between T2DM and cognitive performance at first cognitive measurement with different sets of adjustment for confounders and vascular risk factors. As age is one of the most important risk factors of cognitive impairment, and for easier interpretation, the difference in cognitive performance associated with T2DM was quantified by age equivalent in years, based on the concept of risk advancement periods [22]. The age equivalent was calculated by dividing the regression coefficients of the exposure variable of interest (here: T2DM) by the regression coefficient of age. Fourthly, for the analysis of changes in cognitive performance between first and second cognitive assessment, to adjust for potential practice effects due to repeated administration of the same cognitive function test, the reliable change index (RCI) was calculated by subtracting the mean difference in COGTEL scores between the first and the second measurement from the individual difference in scores and dividing the result by the SD of the difference in those scores [23]. An additional advantage of using the RCI is that the standardization results in directly comparable means and regression coefficients irrespective of the original scales. Finally, generalized linear models were fit to examine the association of T2DM and cognitive decline between both cognitive measurements using the RCI and again calculating the age equivalent.

Missing covariate values were handled by performing multiple imputation. We used the Markov chain Monte Carlo method and built an imputation model containing the following variables (percentage of missing values for incomplete variables is given in brackets): age, sex, education (2.3%), T2DM (2.3%), BMI (1.8%), *APOE* genotype (5.7%), HbA_1c_ (2.3%), history of stroke (2.6%), hypertension, coronary heart disease, depression, smoking status (1.1%), alcohol consumption (2.2%), sleeping disorder (0.4%), hearing impairment, and all subcomponent scores of the COGTEL instrument. The multiple imputation was performed using the MI procedure in SAS. Five multiply imputed datasets were produced, and the procedure MIANALYZE was used to combine those for analysis.

All statistical analyses were carried out using the software SAS version 9.4 (SAS Institute Inc., Cary, N.C., USA). Statistical tests were two-sided. An alpha level of 0.05 was applied for statistical significance testing.

## Results

The main characteristics of the eligible study participants at first COGTEL measurement are shown in Table 1. Both participants with T2DM (*n* = 143; 19.5%) and without T2DM (589; 80.5%) were on average about 74 years old. Relevant differences in the distribution of characteristics were seen for single lifestyle variables and comorbidities. Prevalence of obesity (BMI ≥ 30 kg/m^2^) was more than twice as high in participants with T2DM, but high alcohol consumption was less prevalent. While history of hypertension, stroke, and CHD was more prevalent in the T2DM group, history of depression was less prevalent. Also, the mean baseline HbA_1c_ value for patients with T2DM was higher than that of T2DM-free participants (6.6 vs. 5.6).

**Table 1:** Participant characteristics by T2DM status at first cognitive assessment (5-year follow up of ESTHER cohort, 2005–2007)

	**T2DM**	**T2DM-free**
**N (%)**	**143 (19.5)**	**589 (80.5)**
Age in years, Mean (SD)	74.3 (2.8)	73.7 (2.7)
Sex (N, %)		
Female	75 (52.4)	339 (57.6)
Male	68 (47.6)	250 (42.4)
School education, N (%)		
≤ 9 years	99 (69.2)	379 (64.4)
> 9 years	44 (30.8)	210 (35.6)
BMI^a^, kg/m.^2^, N (%)		
≤ 25	24 (16.8)	190 (32.3)
25–30	65 (45.4)	297 (50.4)
> 30	54 (37.8)	102 (17.3)
APOE e4 carrier, N (%)		
Yes	35 (24.5)	157 (26.7)
No	108 (75.5)	432 (73.3)
HbA1c in %b, Mean (SD)	6.6 (0.9)	5.6 (0.4)
History of stroke, N (%)		
Yes	17 (11.9)	29 (4.9)
No	126 (88.1)	560 (95.1)
History of hypertension, N (%)		
Yes	110 (76.9)	382 (64.9)
No	33 (23.1)	207 (35.1)
History of CHD, N (%)		
Yes	44 (30.8)	130 (22.1)
No	99 (69.2)	459 (77.9)
History of depression, N (%)		
Yes	15 (10.5)	89 (15.1)
No	128 (89.5)	500 (84.9)
Smoking, N (%)		
Never	86 (60.1)	361 (61.3)
Former	50 (35.0)	197 (33.5)
Current	7 (4.9)	31 (5.3)
Alcohol consumption^c^, N (%)		
None	56 (39.2)	173 (29.4)
Low-to-moderate	67 (46.8)	270 (45.8)
High	20 (14.0)	146 (24.8)
Sleeping disorder, N (%)		
Never	30 (21.0)	152 (25.8)
Rarely	36 (25.2)	128 (21.7)
Sometimes	48 (33.5)	187 (31.8)
Most of the time	19 (13.3)	93 (15.8)
Always	10 (7.0)	29 (4.9)
Hearing impairment, N (%)		
Yes	10 (7.0)	36 (6.1)
No	133 (93.0)	553 (93.9)

*CHD* Coronary heart disease, *BMI* Body mass index

^a^BMI < 25: underweight and normal range, 25 ≤ BMI < 30: overweight, BMI ≥ 30: obesity

^b^Measured at ESTHER baseline

^c^Low-to-moderate: Women > 0 to 70 g/week, Men: > 0 to 140 g/week; High: Women ≥ 70 g/week, Men ≥ 140 g/week

Cognitive performance measures by prevalence of T2DM per domain and globally at both COGTEL measurements are shown in Table 2. The mean total COGTEL scores (SD) for patients with T2DM were 27.4 (8.3) at first and 29.4 (8.7) at second COGTEL measurement, and were 1.91 and 1.34 points lower compared to those without T2DM after adjustment for age and sex, with the difference at second COGTEL measurement not reaching statistical significance. At first assessment, participants with T2DM had lower COGTEL scores overall and in all domains except for working memory than T2DM-free participants, with the differences being statistically significant for verbal short-term memory, verbal long-term memory and global cognitive function. At second measurement, participants with prevalent T2DM scored lower in all domains and overall, but the difference was only statistically significant for the verbal fluency domain. Generally, higher COGTEL scores were measured at the second measurement three years after the first measurement for both T2DM and T2DM-free participants, possibly due to well-known practice effects in longitudinal assessment of cognitive function in older adults.

**Table 2:** Means and age- and sex-adjusted mean differences of cognitive performance* according to prevalence of T2DM at first and second cognitive assessment (5-year follow up of ESTHER cohort in 2005–2007 and 8-year follow-up in 2008–2010)

	**First cognitive measurement (*N* = 732)**				**Second cognitive measurement (*N* = 732)**			
	**T2DM**	**T2DM-free**	**B**^**a**^	***P*-value**	**T2DM**	**T2DM-free**	**B**^**a**^	***P*-value**
**N**	**143**	**589**			**185**	**547**		
Prospective memory, N (%)								
0	58 (40.6)	190 (32.3)			72 (38.9)	176 (32.2)		
1	85 (59.4)	399 (67.7)	-0.16	0.092	113 (61.1)	371 (67.8)	-0.14	0.108
Working memory, Mean (SD)	6.6 (2.6)	6.4 (2.5)	0.28	0.237	7.4 (3.0)	7.6 (2.5)	-0.13	0.575
Inductive reasoning, Mean (SD)﻿﻿	3.2 (1.8)	3.6 (1.8)	-0.36	0.044	3.2 (1.6)	3.4 (1.8)	-0.16	0.298
Verbal short-term memory, Mean (SD)﻿	3.9 (1.7)	4.3 (1.9)	-0.41	0.024	4.7 (1.8)	5.1 (1.9)	-0.30	0.070
Verbal long-term memory, Mean (SD)﻿	4.4 (1.6)	4.8 (1.7)	-0.39	0.023	5.4 (1.7)	5.7 (1.7)	-0.17	0.236
Verbal fluency, Mean (SD)﻿	22.0 (6.0)	23.3 (6.0)	-1.14	0.053	21.4 (5.9)	22.8 (6.3)	-1.28	0.022
Global cognitive function (COGTEL score), Mean (SD)﻿	27.4 (8.3)	29.4 (8.7)	-1.91	0.025	30.4 (8.3)	31.9 (8.1)	-1.34	0.062

^*^Higher scores denote better cognitive performance

^a^Mean differences and *P*-values are from multivariate logistic or linear regression models adjusted for age and sex

The results of multivariable adjusted models on the cross-sectional association between T2DM and cognitive performance at first COGTEL measurement are shown in Table 3. Except for the working memory domain, results indicated that presence of T2DM was associated with lower scores (β < 0 in linear regression models) and lower odds of scoring high (OR < 1 in the logistic regression model for prospective memory), with the association being statistically significant for the verbal short-term memory and verbal long-term memory domains in models 1 and 2. Regarding global cognitive function, the models consistently yielded a negative association with T2DM, which however did not reach statistical significance. This difference between T2DM and T2DM-free participants in global cognitive performance translates to an age equivalent of approximately four years when adjusting for all potential confounders and vascular risk factors in model 3.

**Table 3:** Adjusted estimates of the association of prevalence of T2DM with cognitive performance at first cognitive assessment (5-year follow up of ESTHER cohort, 2005–2007, *N* = 732)

	**Regression analysis, adjusted estimates**^**a**^** for COGTEL sub-components and total score**		
	**Model 1**	**Model 2**	**Model 3**
Prospective Memory^b^, OR (95% CI)	0.87 (0.72 to 1.06)	0.87 (0.72 to 1.06)	0.88 (0.72 to 1.08)
Working memory^c^, β (95% CI)	0.34 (-0.13 to 0.81)	0.33 (-0.14 to 0.81)	0.46 (-0.02 to 0.95)
Inductive reasoning^c^, β (95% CI)	-0.27 (-0.61 to 0.06)	-0.27 (-0.61 to 0.06)	-0.17 (-0.52 to 0.18)
Verbal short-term memory^c^, β (95% CI)	-0.36 (-0.71 to -0.01)	-0.36 (-0.71 to -0.01)	-0.29 (-0.65 to 0.07)
Verbal long-term memory^c^, β (95% CI)	-0.34 (-0.67 to -0.02)	-0.35 (-0.67 to -0.02)	-0.27 (-0.61 to 0.06)
Verbal fluency^c^, β (95% CI)	-0.86 (-1.97 to 0.25)	-0.86 (-1.97 to 0.25)	-0.29 (-1.43 to 0.85)
Global cognitive function (COGTEL score)^c^, β (95% CI)	-1.48 (-3.06 to 0.09)	-1.49 (-3.07 to 0.08)	-0.91 (-2.53 to 0.72)
Age equivalent of difference in global cognitive function (years)	6.1	6.1	3.8

^a^Model 1 adjusted for age, sex, education and hearing impairment at first cognitive measurement

Model 2 additionally adjusted for APOE genotype

Model 3 additionally adjusted for BMI, smoking, alcohol consumption, presence of stroke, hypertension, CHD, depression, and sleeping disorder

^b^Results from logistic regression analysis, OR (95% CI) < 1 means T2DM is associated with lower odds of scoring high

^c^Results from multiple linear regression analysis, β (95% CI) < 0 means T2DM is associated with lower cognitive scores

Changes in cognitive performance between COGTEL measurements after adjustment for practice effects through the reliable change index are shown in Table 4. A negative reliable change index denotes a decreased performance, i.e. cognitive decline. The results indicate that the subgroups of participants with prevalent T2DM at first COGTEL measurement had a stronger decline in the COGTEL sub-component and total scores compared to T2DM-free participants after correction for practice effects, with the strongest decline seen in the working memory domain. When considering the T2DM status at second cognitive measurement (i.e., both prevalent and incident T2DM cases), a decline was also particularly observed for the working memory domain.

**Table 4:** T2DM status and change in cognitive performance between first and second cognitive assessment (5-year follow up of ESTHER cohort in 2005–2007 and 8-year follow-up in 2008–2010) using the reliable change index^a^

	**Prevalent T2DM**	**Prevalent and incident T2DM**	**T2DM-free** **(non-prevalent and non-incident T2DM)**
N	**143**	**185**	**547**
Working memory, Mean (SD)	-0.187 (1.09)	-0.146 (1.07)	0.050 (0.97)
Inductive reasoning, Mean (SD)	-0.008 (0.91)	0.018 (0.92)	-0.006 (1.03)
Verbal short-term memory, Mean (SD)	-0.087 (1.00)	-0.002 (0.99)	0.001 (1.00)
Verbal long-term memory, Mean (SD)	-0.055 (1.06)	0.005 (1.06)	-0.001 (0.98)
Verbal fluency, Mean (SD)	-0.082 (1.01)	-0.009 (1.00)	0.003 (1.00)
Global cognitive function (COGTEL score), Mean (SD)	-0.098 (1.03)	-0.017 (1.02)	0.006 (0.99)

^a^Positive RCI-values denote an increase in cognitive performance, negative RCI-values denote a decrease

Table 5 shows the multivariable adjusted results for the longitudinal associations between prevalence of T2DM at 5-year follow-up and the change in COGTEL sub-component and total scores between the two cognitive measurements, corrected for practice effects by using the reliable change index. While a tendency towards a stronger cognitive decline in participants with T2DM was observed for all domains and global cognitive function, the associations were only statistically significant for working memory in all models. The difference in the decline of global cognitive performance between COGTEL measurements, as measured with the COGTEL total score, translates into an age equivalent of about four years when considering full adjustment for potential confounders and vascular risk factors.

**Table 5:** Adjusted estimates of the association of prevalence of T2DM status at 5-year follow-up with change in cognitive performance between first and second cognitive assessment (5-year follow up of ESTHER cohort in 2005–2007 and 8-year follow-up in 2008–2010, *N* = 732) using the reliable change index

	**Generalized linear models, adjusted estimates**^**a**^** for COGTEL scores**		
	**Model 1** **β (95%CI)**	**Model 2** **β (95%CI)**	**Model 3** **β (95%CI)**
Working memory	-0.23 (-0.42 to -0.05)	-0.23 (-0.41 to -0.05)	-0.25 (-0.44 to -0.06)
Inductive reasoning	-0.004 (-0.19 to 0.18)	-0.007 (-0.19 to 0.18)	-0.03 (-0.22 to 0.16)
Verbal short-term memory	-0.11 (-0.29 to 0.08)	-0.11 (-0.29 to 0.08)	-0.14 (-0.33 to 0.04)
Verbal long-term memory	-0.06 (-0.24 to 0.13)	-0.05 (-0.24 to 0.13)	-0.09 (-0.28 to 0.09)
Verbal fluency	-0.08 (-0.27 to 0.10)	-0.09 (-0.27 to 0.10)	-0.10 (-0.28 to 0.09)
Global cognitive function (COGTEL score)	-0.11 (-0.29 to 0.07)	-0.11 (-0.29 to 0.07)	-0.15 (-0.33 to 0.04)
Age equivalent of difference in global cognitive function (years)	3.4	3.4	4.4

^a^Model 1 adjusted for age, sex, education and hearing impairment

Model 2 additionally adjusted for APOE genotype

Model 3 additionally adjusted for BMI, smoking, alcohol consumption, presence of stroke, hypertension, CHD, depression, and sleeping disorder

## Discussion

The present study examined cognitive performance over a 3-year interval using a comprehensive neuropsychological instrument to investigate the association between presence of T2DM and both cognitive performance and cognitive decline in a cohort of older adults in Germany. The difference in scoring in the global cognition test is substantial, as it is equivalent to the cognitive decrement associated with a four-year age difference. The findings presented support previous findings that T2DM is associated with worse global cognitive performance as well as in memory-related domains (verbal short-term and long-term memory). After adjusting for demographic variables and *APOE* genotype, the associations with verbal short-term and long-term memory remained statistically significant, but they were attenuated and no longer statistically significant after additional adjustment for other vascular risk factors of cognitive impairment. While this finding might reflect a lack of statistical power, it is also possible that the association of T2DM with cognitive performance is mediated by vascular factors. Our study also indicated that prevalent T2DM is associated with a stronger decline in global cognitive performance, in memory-related domains and in verbal fluency over three years. However, a statistically significant association of T2DM with cognitive decline was detected only for the working memory domain.

Several studies have investigated the association between T2DM and cognitive performance. Our findings are consistent with most of them showing that T2DM is a predictor of worse cognitive performance in multiple domains [15, 24, 25]. Several potential biological explanations have been proposed for the association with cognitive impairment, such as T2DM-related cerebral microvascular and macrovascular damage, both of which have been suggested to contribute to cognitive decrements [26–28]. The exact pathways are however yet unclear. A recent meta-analysis found regional cerebral hypoperfusion in T2DM to be associated with a wide range of cognitive disorders, suggesting that decreased cerebral blood flow in T2DM possibly due to chronic inflammation may be a potential cause of cognitive impairment [29]. Another explanation of the link between T2DM and cognition pertains to the role of brain insulin resistance [30]. After adjustment for potential vascular mediators, the association between T2DM and cognitive function was attenuated in our study but did not disappear, suggesting that T2DM is associated with cognition independently from the vascular risk profile.

This study particularly confirmed a positive association between T2DM and worse performance on the memory domain after appropriate confounder adjustment. Consistent with prior findings [25], results presented in this study indicate that the memory domain is more sensitive to performance decrements in people with T2DM compared to T2DM-free participants. Two meta-analyses also detected a significant association between T2DM and worse performance in the executive function domain as assessed with verbal fluency tasks [25, 31]. In this sample, we only detected a non-significant tendency in participants with T2DM towards worse performance and stronger decline in verbal fluency after confounder adjustment, suggesting that vascular factors mostly explain the association. It should be noted however, that in clinical practice, the question of whether such cognitive deficits are a consequence of T2DM or of associated vascular factors is a rather theoretical one. Cognitive deficits in T2DM patients may have major implications because they decrease T2DM patients’ self-care capabilities by interfering with their glucose self-monitoring abilities and their medication, diet and exercise adherence, and hence potentially adversely impact their prognosis [32–34]. Hence, close attention should be paid to even subtle cognitive deficits to identify those with higher risk of cognitive impairment to prevent deterioration and reduce the T2DM-related disease burden.

In contrast to the consistent results from a large number of studies that confirmed a relation between T2DM and worse cognitive performance, less evidence regarding the longitudinal relationship between T2DM and accelerated cognitive decline is available, and results thus far are somewhat heterogeneous [9], with several studies having failed to find a significant association between T2DM and accelerated cognitive decline (in reference to T2DM-free subjects) in the older population [14–16]. In accordance with these mixed results, we found a non-significant tendency towards an association between T2DM status and decline of global cognitive function, and found a statistically significant association only for the working memory domain. Potential explanations of such variations in findings point to the importance of the onset of cognitive changes related to T2DM. A recent review hypothesized that microvascular disease processes leading to brain disorders might start long before T2DM-onset (coined as the “ticking clock hypothesis”), i.e., at pre-clinical stages in middle age [27]. This might explain why we rather observed baseline differences in cognitive performance according to T2DM status than differences in cognitive decline in this study. It is possible, that in our older sample (≥ 70 years old on average), age has become the dominant determinant of cognitive decline, and T2DM-related cognitive decline from middle age is then only reflected in baseline differences in cognitive performance. Furthermore, the ticking clock hypothesis also implies that such processes affect some T2DM-free participants in yet undetected pre-clinical stages as well, potentially diluting the association between T2DM and cognitive decline. Finally, anti-diabetic medication might also influence the cognitive status of persons with T2DM; particularly metformin, glucagon-like peptide-1 (GLP-1) receptor agonists and dipeptidyl peptidase-4 inhibitors have been suggested to potentially exert neuroprotective effects [35].

Some limitations should be noted in relation to the findings of this study. First, our study had limited power to detect modest associations between T2DM and cognitive function due to the small sample size of several subgroups and the rather short follow-up time of three years. Second, potential selection bias may exist due to sample attrition related to T2DM-related morbidity and mortality. We did not consider or collect the information of people who died or who were lost to follow-up, and since failing to participate in follow-up measurements itself could well be linked to worse cognitive function or to T2DM-related conditions, our results may underestimate the association of T2DM with cognitive decline. Third, we may have missed individuals at pre-clinical T2DM stages, a crucial time when cognitive decrements may occur. Therefore, we may have again underestimated the effects of T2DM on cognition. Finally, we cannot rule out residual confounding due to unmeasured or imperfect measurement of confounders.

The primary strength of the present study is that we investigated the long-term association between T2DM and cognitive function using both a cross-sectional and longitudinal design and a comprehensive neuropsychological instrument. Also, we calculated the reliable change index to adjust for practice effects due to repeated testing to measure cognitive change instead of using raw mean differences. Additionally, potential bias from missing values in covariates was minimized by applying multiple imputation.

In summary, this study suggests that T2DM is associated with worse cognitive performance in a global cognitive test equivalent to the decrement in global cognition of around 4 years of aging in an older population in Germany, with the memory component being particularly affected. In a longitudinal perspective, a significant association between T2DM and cognitive decline was found for the working memory domain. Future studies should try to clarify the mechanism of onset and the timing of T2DM-related cognitive decrements as well as the role of anti-diabetic medication in limiting the progression of cognitive decline. Large-scale longitudinal studies with younger samples or pre-diabetic and newly diagnosed subjects are needed to ascertain the relation between pre-clinical stage and T2DM-related cognitive decline. Our findings also underline the need for studies investigating how cognitive deficits in T2DM patients affect self-management, and how to thwart potential adverse effects.

## Supplementary Information

**Additional file 1: Supplemental Table. **Comparison of participant characteristics of participants with repeated COGTEL assessment with participants lost to the second COGTEL assessment (5-year follow up of ESTHER cohort in 2005-2007 and 8-year follow-up in 2008-2010).

## Data Availability

The datasets generated and/or analysed during the current study are not publicly available due to conflicts with study-related data protection regulations but are available from the study centre (b.schoettker@dkfz.de) on reasonable request.
