# Supplementary material for: Type 2 diabetes mellitus and cognitive decline in older adults in Germany – results from a population-based cohort
Source: BMC Geriatr. 2022 May 26;22:455. doi: 10.1186/s12877-022-03151-y (PMC9137064; doi:10.1186/s12877-022-03151-y)
Supplement: Supplementary file 1 — Additional file 1: Supplemental Table. Comparison of participant characteristics of participants with repeated COGTEL assessment with participants lost to the second COGTEL assessment (5-year follow up of ESTHER cohort in 2005-2007 and 8-year follow-up in 2008-2010). [file 12877_2022_3151_MOESM1_ESM.pdf]

Supplemental Table. Comparison of participant characteristics of participants with repeated COGTEL assessment with participants lost to the second COGTEL assessment (5-year follow up of ESTHER cohort in 2005-2007 and 8-year follow-up in 2008-2010)

	**Participants with repeated COGTEL assessment**	**Participants lost to second COGTEL assessment**
**N (%)**	**777**	**1147**
Age in years, Mean (SD)	73.8 (2.7)	74.2 (2.9)
Prevalence of T2DM (N, %)		
Yes	160 (21.0)	310 (27.4)
No	600 (79.0)	823 (72.6)
Missing	17	14
Sex (N, %)		
Female	437 (56.2)	653 (56.9)
Male	340 (43.8)	494 (43.1)
School education, N (%)		
≤ 9 years	505 (66.6)	875 (79.3)
>9 years	253 (33.4)	228 (20.7)
Missing	19	44
BMI[a], kg/m^2, N (%)		
≤ 25	222 (29.0)	300 (26.7)
25-30	378 (49.5)	533 (47.5)
>30	164 (21.5)	290 (25.8)
Missing	13	24
APOE e4 carrier, N (%)		
Yes	177 (24.1)	279 (25.5)
No	557 (75.9)	813 (74.5)
Missing	43	55
HbA_{1c} in %[b], Mean (SD)	5.8 (0.7)	5.9 (0.9)
Missing	17	13
History of stroke, N (%)		
Yes	48 (6.4)	96 (8.8)
No	706 (93.6)	992 (91.2)
Missing	23	59
History of hypertension, N (%)		
Yes	522 (67.2)	834 (72.7)
No	255 (32.8)	313 (27.3)
History of CHD, N (%)		
Yes	187 (24.1)	294 (25.6)
No	590 (75.9)	853 (74.4)
History of depression, N (%)		
Yes	115 (14.8)	195 (17.0)
No	662 (85.2)	952 (83.0)
Smoking, N (%)		
Never	470 (61.1)	663 (57.9)
Former	257 (33.4)	392 (34.3)
Current	42 (5.5)	89 (7.8)
Missing	8	3
Alcohol consumption[c], N (%)		
None	237 (31.3)	468 (41.9)
Low-to-moderate	353 (46.6)	447 (40.0)
High	168 (22.1)	203 (18.1)
Missing	19	29

Sleeping disorder, N (%)		
Never	193 (24.9)	343 (30.1)
Rarely	174 (22.5)	186 (16.3)
Sometimes	247 (31.9)	340 (29.8)
Most of the time	116 (15.0)	190 (16.7)
Always	44 (5.7)	81 (7.1)
Missing	3	7
Hearing impairment, N (%)		
Yes	55 (7.1)	112 (9.8)
No	722 (92.9)	1035 (90.2)

CHD = Coronary heart disease; BMI = Body mass index.

[a] BMI<25: underweight and normal range, 25≤BMI<30: overweight, BMI≥30: obesity

[b] Measured at ESTHER baseline

[c] Low-to-moderate: Women >0 to 70g/week, Men: >0 to 140g/week; High: Women ≥70g/week, Men ≥140g/week
